# Ginsenosides: changing the basic hallmarks of cancer cells to achieve the purpose of treating breast cancer

**DOI:** 10.1186/s13020-023-00822-9

**Published:** 2023-09-25

**Authors:** Rui-yuan Jiang, Zi-ru Fang, Huan-ping Zhang, Jun-yao Xu, Jia-yu Zhu, Ke-yu Chen, Wei Wang, Xiao Jiang, Xiao-jia Wang

**Affiliations:** 1 Zhejiang Cancer Hospital, Hangzhou Institute of Medicine (HIM), Chinese Academy of Sciences, Hangzhou, 310022 Zhejiang China; 2 Zhejiang Chinese Medical University, NO. 548, Binwen Road, Binjiang District, Hangzhou, 310000 Zhejiang China; 3 Wenzhou Medical University, No. 270, Xueyuan West Road, Lucheng District, Wenzhou, 325027 Zhejiang China; 4 Department of Basic Medical Sciences, Guangxi University of Chinese Medicine, NO. 13, Wuhe Road, Qingxiu District, Nanning, 530022 Guangxi China

**Keywords:** Ginsenoside, Breast cancer, Hallmarks of cancer, Apoptosis, Epithelial-mesenchymal transition, Cell growth

## Abstract

In 2021, breast cancer accounted for a substantial proportion of cancer cases and represented the second leading cause of cancer deaths among women worldwide. Although tumor cells originate from normal cells in the human body, they possess distinct biological characteristics resulting from changes in gene structure and function of cancer cells in contrast with normal cells. These distinguishing features, known as hallmarks of cancer cells, differ from those of normal cells. The hallmarks primarily include high metabolic activity, mitochondrial dysfunction, and resistance to cell death. Current evidence suggests that the fundamental hallmarks of tumor cells affect the tissue structure, function, and metabolism of tumor cells and their internal and external environment. Therefore, these fundamental hallmarks of tumor cells enable tumor cells to proliferate, invade and avoid apoptosis. Modifying these hallmarks of tumor cells represents a new and potentially promising approach to tumor treatment. The key to breast cancer treatment lies in identifying the optimal therapeutic agent with minimal toxicity to normal cells, considering the specific types of tumor cells in patients. Some herbal medicines contain active ingredients which can precisely achieve this purpose. In this review, we introduce Ginsenoside's mechanism and research significance in achieving the therapeutic effect of breast cancer by changing the functional hallmarks of tumor cells, providing a new perspective for the potential application of Ginsenoside as a therapeutic drug for breast cancer.

## Introduction

In 2021, breast cancer became the most common malignancy in women worldwide, accounting for more than 30% of the total number of newly diagnosed cancers. According to the American Cancer Society, about 281,500 women were diagnosed with breast cancer in 2021 in the United States, resulting in 43,600 deaths. Predictive modeling suggests that around 287,850 new cases of breast cancer and approximately 43,250 deaths are projected in the United States in 2022 [1]. The high incidence and number of deaths caused by breast cancer impose a severe economic burden on countries worldwide, placing immense importance on ensuring public safety and well-being.

**Fig. 1:**
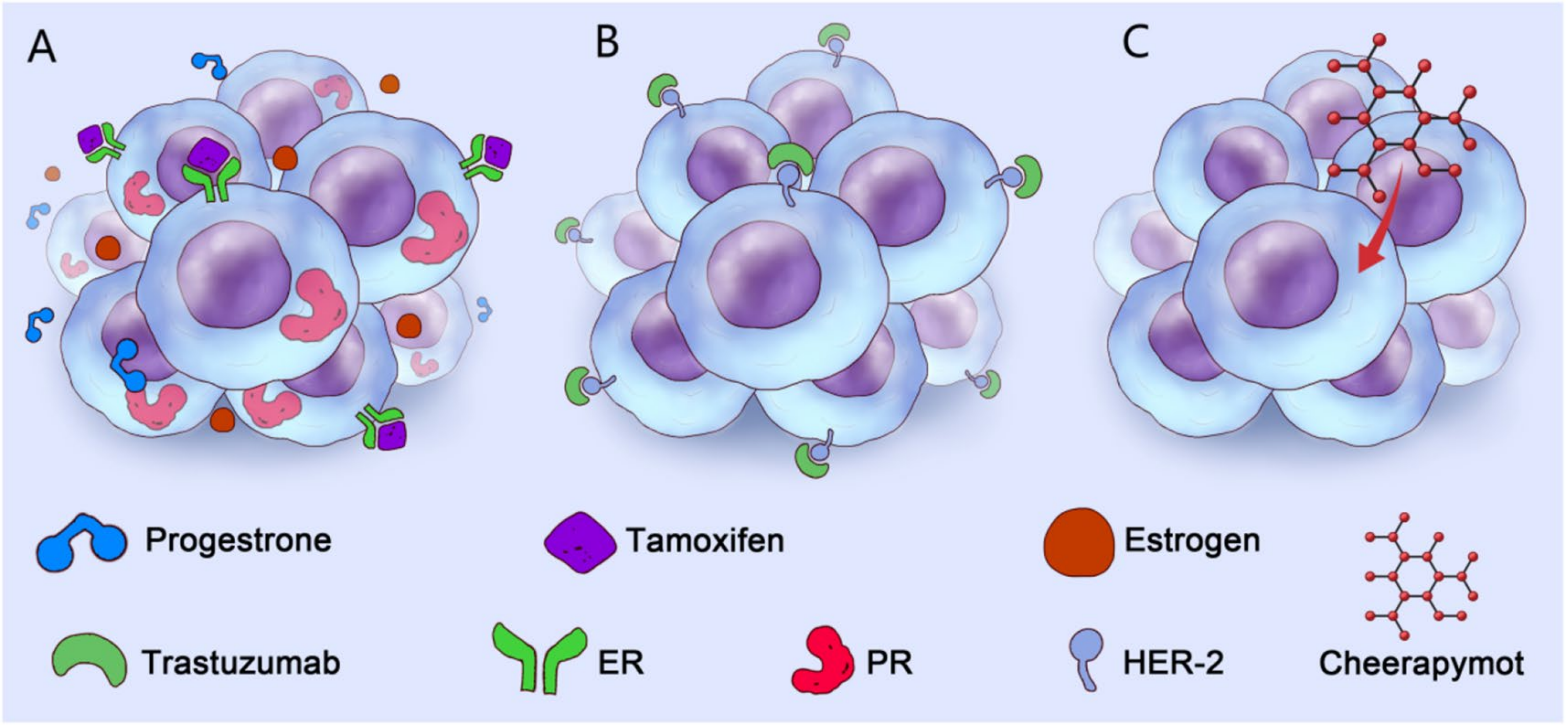
Molecular typing and treatment of breast cancer. The current molecular typing of breast cancer is mainly based on the surface receptors of the breast cancer cell membrane, which is mainly divided into luminal type (**A**), HER2 type (**B**), and Triple-negative type (**C**). At present, due to the expression of hormone receptors on the membrane surface, anti-hormone therapy (AI, TAM, CDK4/6i) is indicated for luminal breast cancer. The primary treatment of HER2 breast cancer is targeted anti-HER2 monoclonal antibody therapy (HER2-TKI, HER2-ADC, HER2 inhibitor). Triple-negative breast cancer can also be divided into six categories (BL1, BL2, IM, LAR, M, MSL, MSL) according to the different activation characteristics of intracellular signaling pathways. Chemotherapy is the main treatment choice for triple-negative breast cancer. However, with the deepening of research, some new anti-tumor drugs (PI3K inhibitors, PD-1 inhibitors, Trop2-ADC, anti-angiogenic drugs) also show the dawn in the clinical treatment of triple-negative breast cancer

It has been found that breast cancer can be classified into three main groups according to whether or not the patient's breast cancer cells express estrogen receptor/progesterone receptor, human epidermal growth factor 2 receptor (HER2) [2]: ER- or PR- positive (also known as luminal type breast cancer), HER2 positive cancer and triple-negative breast cancer (TNBC) (ER-/PR-/HER2-). Among them, luminal B breast cancer can be divided into two main categories, HER2 positive and HER2 negative, based on the presence or absence of positive expression of HER2. It is generally believed that triple-negative breast cancer has large heterogeneity with large differences in cell molecular types. However, Lehmann et al. [3] performed a cluster analysis of 21 groups of gene expression profiling data in 587 cases of TNBC. They found that TNBC could be further classified into six subtypes, including basal-like type 1 (BL1), basal-like type 2 (BL2), immune modulatory type (IM), luminal androgen receptor type (LAR), mesenchymal type (M), and mesenchymal stem-like cell type (MSL). These six subtypes represent distinct molecular features of TNBC and different therapeutic strategies (Fig. 1, Table 1).

**Table 1:** Molecular typing of breast cancer

Molecular typing (Molecular subtype)	Molecular characteristics					Possible treatment strategies	Cell line names represented
	HER2	ER	PgR	Ki67	Other molecular features		
HER2 positive	Positive	–	–	Whatever	/	Anti-Her2 treatment	SK-BR-3, AU-565, MDA-MB-453
Luminal A	–	Positive	Positive and high expression of	Low expression	/	Endocrine therapy	MCF-7, T47D, SUM185
Luminal B (HER2 negative)	–	Positive	Low expression or -	High expression	/	Endocrine therapy combined with chemotherapy	/
Luminal B (HER2 positive)	Positive	Positive	Whatever	Whatever	/	Anti-Her2 treatment with endocrine therapy combined	BT-474, ZR-75-1
Triple-negative	–	–	–	Whatever	/	Chemotherapy based comprehensive treatment	MDA-MB-468, SUM190, BT549, MDA-MB-231, Hs578T, SUM1315, BT483,MCF-12A, HBL101, HS598T (Appropriate cell lines were selected based on different populations and genetic circumstances)
Triple-negative (BL1)	/	/	/	/	Highly expressed cell cycle and DNA damage response genes	Platinum based chemotherapeutics, PARP inhibitors	
Triple-negative (BL2)	/	/	/	/	Increased activity of growth factor pathways and PI3K pathway	mTOR inhibitors, growth factor inhibitors	
Triple-negative (IM)	/	/	/	/	Highly expressed immune-related genes	Immune checkpoint inhibitors	
Triple-negative (LAR)	/	/	/	/	AR signaling pathway activation; enrichment of PIK3CA mutations	Antiandrogens, PI3K inhibitors	
Triple-negative (M)	/	/	/	/	EMT characteristics; growth factor pathway activation	mTOR inhibitors, growth factor inhibitors	
Triple-negative (MSL)	/	/	/	/	EMT characteristics; growth factors and PI3K pathway activation	mTOR inhibitors, PI3K inhibitors, growth factor inhibitors	

In clinical practice, breast cancer cells in patients are classified and treated based on various factors, including tumor growth type (in situ or invasive) and specific classifications. Treatment interventions typically involve a combination of surgical resection, radiation therapy, immunotherapy, targeted therapy, and chemotherapy [4, 5]. However, determining the optimal treatment for patients is often quite challenging because of the difficulty in determining the appropriate dose of therapeutic drugs for different patients and the optimal surgical resection margin. Moreover, different therapeutic agents can adversely affect normal tissue cells, leading to reduced bone mineral density, immune-related inflammation, and diarrhea, all of which negatively impact the patient’s quality of life. And the effects that different therapeutic agents bring to normal tissue cells, such as reduced bone density, immune related inflammation and diarrhea, all give a reduced quality of life of patient [6]. Additionally, patients may experience ineffective treatment if the therapeutic drug dosage needs to be reduced due to intolerance to side effects, resulting in suboptimal therapeutic levels [7]. As research in breast cancer-related molecular biology advances, an increasing number of molecular mechanisms that contribute to the proliferation and invasion of breast cancer are being discovered. This research has led to the development of more small-molecule drugs that specifically induce the death of breast cancer cells while sparing normal cells, offering targeted treatment options for breast cancer [8]. Additionally, experiments have demonstrated the promising potential of various active ingredients found in natural products to treat breast cancer [9]. Further exploration of these natural compounds has revealed their ability to partially replace chemotherapy drugs in killing tumor cells. Moreover, they can help reduce the side effects associated with chemotherapy, enhance the efficacy of tumor cell eradication, and potentially lower the required doses of chemotherapeutic drugs used in clinical practice [10].

The fundamental characteristics of tumor cells encompass their distinct biological capabilities acquired during the progressive transformation from normal cells to tumor cells [11]. These characteristics serve as a framework for understanding the intricacies of neoplastic diseases and account for the significant heterogeneity observed among tumor cells. The specific biological abilities of tumor cells consist of fourteen fundamental traits, including sustaining proliferative signals, evading growth inhibitory factors, resisting cell death, and achieving replicative immortality. These hallmarks are underpinned by genomic instability, which further contributes to the growing genetic diversity observed among tumor cells. Consequently, tumor cells exhibit increasingly disparate hallmarks compared to normal cells [12]. The hallmarks of tumor cells play a vital role in maintaining tumor survival and development conditions and promoting their growth and development. Currently, research on tumor treatment extends beyond focusing solely on tumor cells and encompasses the various components within the tumor microenvironment. The aim is to induce tumor cell apoptosis by targeting and inhibiting the fundamental characteristics of tumors. It has been established that the proliferation, invasion, metastasis, immune evasion, and angiogenesis of tumor cells are outcomes of the intricate interplay between tumor cells and the tumor microenvironment. These processes are considered vital hallmarks of tumor cells [13]. However, targeting one or more of the basic hallmarks of tumors can effectively inhibit tumor angiogenesis, inhibit tumor release of pro-inflammatory mediators, and so on, to induce tumor apoptosis [14]. At present, the most commonly used anti-angiogenic drugs in the clinic are drugs targeted at inhibiting the angiogenesis of tumor tissues and inducing tumor cell apoptosis. For instance, anti-angiogenic drugs can block neoendothelial angiogenesis necessary for tumor cell proliferation by inhibiting various angiogenic factors such as VEGF and PDGF and cause tumor cells to lack the nutrients required for growth to induce apoptosis [15]. There is a rich literature substantiating the ability of Radix Ginseng and its active components to effectively modify the fundamental characteristics of tumor cells, thereby achieving therapeutic effects in tumor treatment [16].

Over the years, significant progress has been achieved in identifying and understanding the active components present in Radix Ginseng. These bioactive components encompass a variety of compounds, including saponins, polysaccharides, flavonoids, volatile oils, and fatty acids [17]. Ginsenosides, which are the most abundant and diverse active compounds in Radix Ginseng, have received considerable attention for their potential in the treatment of breast cancer. They have been extensively studied and reported for their anti-tumor properties, immune-enhancing effects, and stress-relieving capabilities [18]. This manuscript provides a comprehensive review of the mechanism and research significance of ginsenosides in treating breast cancer by modulating the tumor microenvironment. Our findings offer a novel perspective on the potential therapeutic application of ginsenosides as a valuable drug in breast cancer treatment.

## The basic hallmarks of tumor cells are necessary for the survival of breast cancer cells

**Fig. 2:**
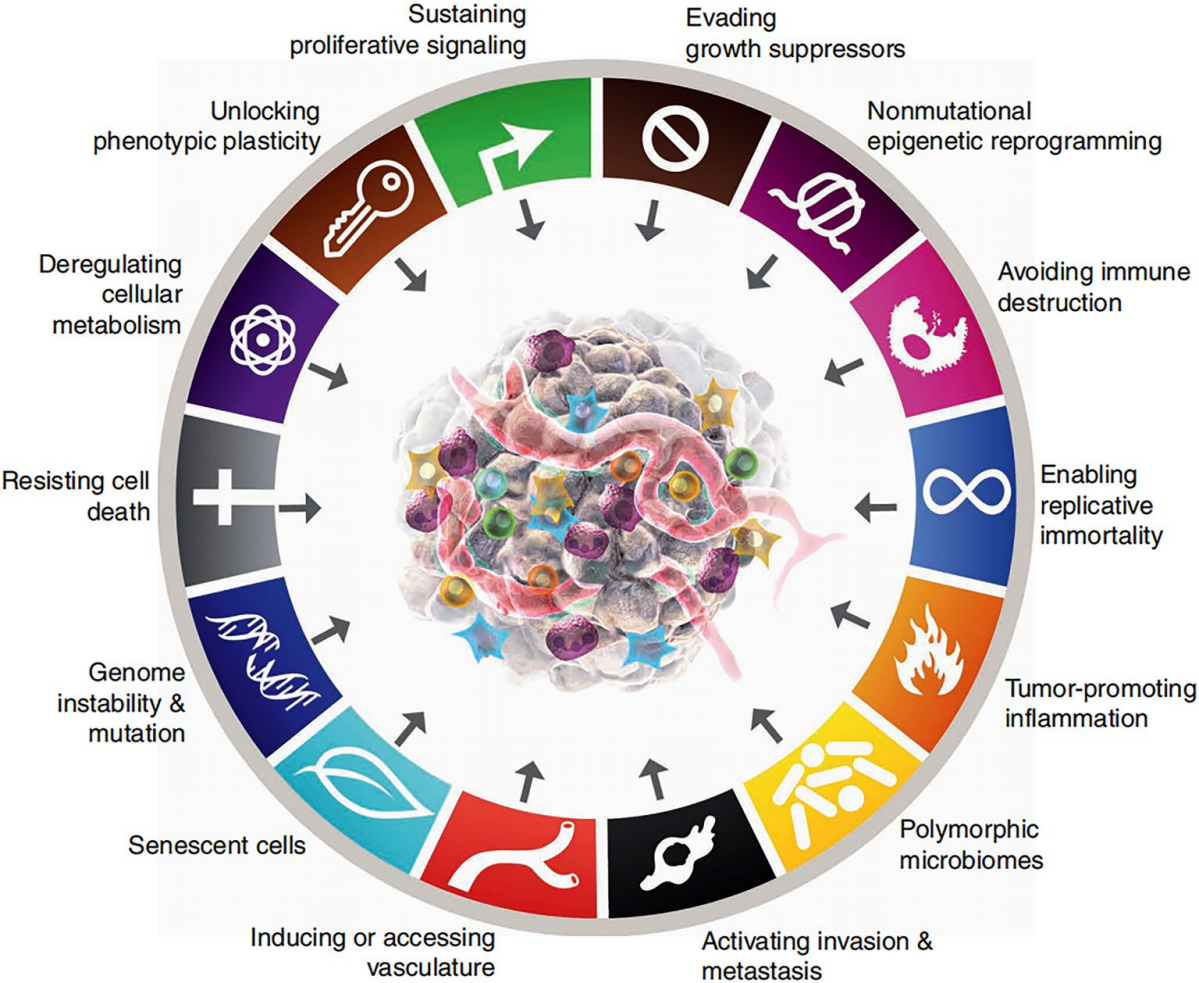
Schematic of Hallmarks of cancer. Hallmarks of Cancer is a process of continuous discovery. Currently, 14 Hallmarks of Cancer have been found: sustaining proliferative signaling, Evolving growth suppressors, Deregulating cellular metabolism, Avoiding immune destruction, Resisting cell death, Enabling replicative immortality, Genome instability & mutation, Tumor-promoting inflammation, Inducing or accessing vascular, Activating invasion & metastasis, Unlocking phenotypic plasticity, Nonmutational epigenetic reprogramming, Senescent cells, Polymorphic microbiomes. The first version of Hallmarks of Cancer was proposed in 2000; six basic Hallmarks of cancer were proposed at that time (Sustaining proliferative signaling, Evolving growth suppressors, Resisting cell death, Enabling replicative immortality, Inducing or accessing vascular, Activating invasion & metastasis). In 2011, four basic Hallmarks were added to the Hallmarks of Cancer (Avoiding immune destruction, Genome instability & mutation, Tumor-promoting inflammation, and Deregulating cellular metabolism). In 2022, four basic Hallmarks were added again (Senescent cells, Polymorphic microbiomes, Unlocking phenotypic plasticity, Nonmutational epigenetic reprogramming). In the future, more Hallmarks of Cancer will be found and added, which will help cancer researchers to continuously discuss and experimentally elaborate relevant treatments for cancer

The fundamental hallmark features of cancer encompass a series of functional capabilities that human cells acquire during their transition from a normal state to tumor growth. These functional abilities are crucial for tumor cells to develop into malignant tumors. Breast cancer cells possess these functional capabilities acquired during the growth phase of malignant tissues. Three versions of Hanahan’s classic reviews on cancer features totaling 14 basic cancer features have been published, and these reviews have become wind vanes in basic cancer research and clinical research (Fig. 2) [12, 19, 20]. The basic hallmarks of cancer summarized so far include: Sustaining proliferative signaling, Evolving growth suppressors, Deregulating cellular metabolism, Avoiding immune destruction, Resisting cell death, Enabling replicative immortality, Genome instability & mutation, Tumor-promoting inflammation, Inducing or accessing vascular, Activating invasion & metastasis, Unlocking phenotypic plasticity, Nonmutational epigenetic reprogramming, Senescent cells, Polymorphic microbiomes. The fundamental hallmarks of breast cancer are a response to the complexity of the pathogenesis of breast cancer, but these characteristics still cannot accurately explain the complex pathogenesis of breast cancer. The precise molecular and cellular mechanisms underlying the development and acquisition of these aberrant phenotypic capabilities during the progression of breast cancer have been found to originate from pre-existing breast cancer progenitor cells. These mechanisms have evolved and acquired over time, leading to the manifestation of these capabilities in breast cancer cells [21]. Like other tumor cells, breast cancer cells do not have these basic hallmarks during the early stages of tumor development. Breast cancer cells also acquire these malignant features gradually during malignant progression [22]. In advanced breast cancer, cancerous cells possess comprehensive abilities to regulate abnormal phenotypes, posing a greater challenge for effective eradication [23]. However, it has been found that various ginsenosides can play a single or comprehensive targeted therapeutic role according to different characteristics of breast cancer [24]. Therefore, ginsenoside and its metabolic components hold significant potential in relevant fields focused on targeting the fundamental characteristics of breast cancer cells to treat breast cancer [25].

## Ginsenoside is the main active ingredient of Radix Ginseng, exhibiting anti-breast cancer effects

Radix Ginseng is the dried root or rhizome of the Araliaceae plant ginseng, officially known as “*Panax ginseng C. A. Meyer*” [26]. In East Asia, Radix Ginseng has long been used to treat and prevent various diseases mainly manifested as body energy deficiency. Approximately 2500 years ago, the first book in China was written, documenting the usage of ginseng. The records indicate that the primary function of Radix Ginseng is its tonic effect, which can nourish the body and provide energy [27]. These beneficial effects can be attributed to its active components, with ginsenoside and its metabolites being the predominant substances found in Radix Ginseng. These components are considered the primary active constituents responsible for the pharmacological effects of Radix Ginseng. Ginsenoside, a water-soluble sterol compound, is commonly referred to as “saponin” because it produces a long-lasting soap-like foam when shaken, which persists even after heating [28].

**Fig. 3:**
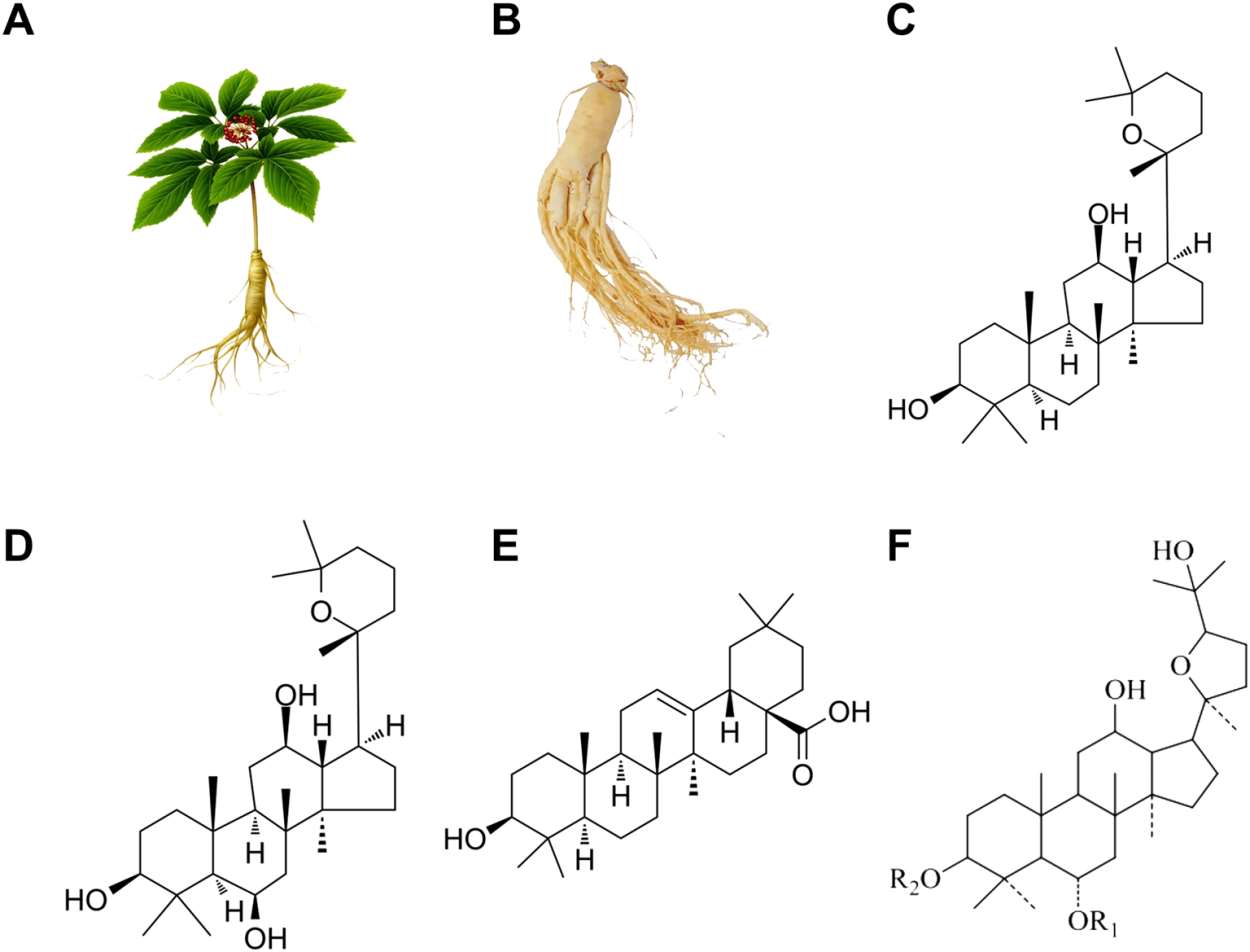
Formula of four chemical structures of ginsenoside, *Panax ginseng C. A. Meyer* plant morphology, and *Panax ginseng C. A. Meyer* medicinal part. **A** *Panax ginseng C. A. Meyer* plant morphology; **B** the main medicinal part of *Panax ginseng C. A. Meyer*—Morphological diagram of *Panax ginseng C. A. Meyer*; **C** a chemical structure diagram of Protopanaxadiol-type ginsenoside; **D** a chemical structure diagram of Protopanaxatriol-type ginsenoside; **E** a chemical structure diagram of Oleanolic Acid-type ginsenoside; **F** a chemical structure diagram of Ocotillol-type ginsenoside

It is now understood that ginsenoside has a dammarane triterpenoid structure. Currently, ginsenoside can be divided into four categories according to the number of steroid backbone and attached glycosyl or hydroxyl groups (Fig. 3, Table 2) [29]. The protopanaxadiol-type ginsenoside comprises ginsenoside Ra1, Ra2, and Ra3, while protopanaxatriol-type ginsenoside includes ginsenoside Re, Rf, and Rg1. Oleanolic Acid-type ginsenoside consists of ginsenoside R0, Rh3, and R1, while Ocotillol-type ginsenoside includes ginsenoside F11. At present, research on Radix Ginseng has revealed that most ginsenosides contained in Radix Ginseng belong to protopanaxadiol type saponin and protopanaxatriol type saponin, while oleanolic acid type ginsenoside accounts for only a smaller proportion [18]. Current evidence suggests that most ginsenoside protopanaxadiol-type and protopanaxatriol-type saponins have definite anti-breast cancer activity [25]. Besides, laboratory studies have confirmed that besides protopanaxadiol and protopanaxatriol, the secondary metabolic derivatives of ginsenoside yield a significant anti-breast cancer effect. Furthermore, laboratory studies have confirmed that secondary metabolic derivatives of ginsenosides, apart from the protopanaxadiol-type and protopanaxatriol-type, also considerably impact anti-breast cancer properties [30]. Although different ginsenosides exhibit varying pharmacological actions against breast cancer, the molecular signals and mechanisms of action differ among the different ginsenosides, rendering the molecular mechanism complex. Consequently, developing ginsenoside preparations with anti-breast cancer effects has become an important aspect of ginsenoside commercialization.

**Table 2:** Structural classification of ginsenosides

Structure type	Ginsenoside specific name	References
Panaxadiol	Compound K	[130, 159]
	Rh2	[66, 89, 103, 115, 116, 118, 119, 132, 150, 157, 169]
	Rg3	[58, 95, 101, 102, 113, 114, 117, 131, 147, 148, 156]
	Rb1	[78, 79]
	Rg5	[60, 61]
	Rk1	[62, 63]
	Rd	[94, 158]
	20(S)-PPD	[160]
	Rg6	[91]
	20(S)-Rh3	[59]
	1f	[69]
	Rb3	[149]
Panaxatriol	Ginsenoside panaxatriol	[49]
	Rh1	[70–72]
	Rg2	[75, 76, 167, 168]
	Rh4	[68]
	Rg1	[64, 65, 90, 120, 170]
	20(S)-PPT	[77]

Ginsenosides can be divided into four categories according to different chemical structures. But there is no indication that the different structures of ginsenosides will affect their treatment in different molecular types of breast cancer. Different types of ginsenosides have different steroid skeletons and the number of attached sugar groups or hydroxyl groups, but the main body of ginsenosides is still dammarane triterpenoid structure. Therefore, the next research direction should focus on the related research of the biological impact of different chemical structures of ginsenosides on different molecular types of breast cancer.

## Metabolic shift and pharmacokinetic changes of ginsenosides in vivo

Given that ginsenosides represent major active components in traditional Chinese Radix Ginseng, oral administration remains the predominant route for their administration until other routes are proven effective and safe [31]. Indeed, when conducting in vivo experiments with ginsenosides, it is crucial to consider their pharmacokinetics. Although studies on the pharmacokinetics of ginsenosides have primarily focused on in vitro cellular models, animal models, and healthy human volunteers [32], the most active research areas are in vitro cellular models and animal models. However, it remains uncertain whether the metabolism of different ginsenosides differs significantly. The general understanding of ginsenoside metabolism is that, after oral administration, ginsenosides undergo partial or complete hydrolysis under acidic conditions by gastric and intestinal microflora, producing active ingredients [33]. The most well-studied ginsenoside, Rg3 and other protopanaxadiol ginsenosides undergo deglycosylation upon metabolism by anaerobic intestinal bacteria. On the other hand, compound K, the end metabolite of some ginsenosides, can be detected in human plasma as early as seven hours after ingestion [34]. In vitro studies have shown that Rg3 in ginsenosides interacts with cytochrome P450 isoenzymes, indicating that some ginsenosides may affect the metabolism of normal anti-tumor drugs. Ginsenoside Rg3 from Radix Ginseng was found to have a weak inhibitory effect on CYP3A4, a moderate inhibitory effect on CYP2C19, CYP1A2, and a potent inhibitory effect on CYP2D4 [35]. Therefore the possible effects of ginsenosides on their production need to be considered when using drugs metabolized via these isozymes. Gut flora is also very important for the metabolism of ginsenosides. Current evidence suggests that some ginsenosides are transformed into other types of ginsenosides after decomposition by the gut flora. For example, ginsenoside Rg3 can be transformed into ginsenoside Rh2 by incubation with intestinal flora in vivo [36]. Although in vitro studies have shown that deglycosylation is one of the major pathways for ginsenoside Rg3 metabolism, studies have not found ginsenosides with complete deglycosylation in animals [37]. The effects of different modes of administration on the metabolic half-life of ginsenosides are highly heterogeneous. When ginsenoside Rg3 was administered intravenously in rats, its half-life was 14 min [38] and 18.5 min [39] in separate studies. The variations in the metabolic time of ginsenoside Rg3 in rats can be attributed to the dissimilar solubility of ginsenoside Rg3 and heterogeneity in its absorption and utilization within rats. These studies overlap in their assertion that ginsenoside Rg3 has high metabolic clearance and low bioavailability when administered intravenously in rats. However, it is important to consider that species differences between rats and humans may influence these findings. In healthy humans, the plasma half-life of ginsenoside Rg3 can reach up to 8 h [40] and 216 h [41] following oral and intramuscular administration, respectively. These results suggest that ginsenoside Rg3 exhibits a longer half-life and potentially higher bioavailability in humans compared to rats. Nevertheless, oral administration remains the most predominant mode of administration of ginsenosides in clinical studies and basic research at present.

The tissue distribution concentration of a drug is a critical factor influencing the treatment of breast cancer with ginsenosides after oral administration [42]. Following the oral administration of ginsenoside 20(S)-Rg3 to rats at a dosage of 68 mg/kg, the concentration of 20(S)-Rg3 in gastrointestinal tissues was the highest, suggesting its significant impact on the digestive tract. However, 20(S)-Rg3 concentrations in other tissues such as muscle, spleen, lung, and adipose were similar to or lower than plasma concentrations. Additionally, 20(S)-Rg3 trace amounts were detected in the brain, heart, and kidney [42]. This illustrates that through oral administration, ginsenoside 20(S)-Rg3 may exert effects on tissues throughout the body. Interestingly, when 20(R)-Rg3 was administered using various routes of administration, its accumulation was predominantly observed in the liver and gastrointestinal tract, without its presence detected in plasma, suggesting that oral administration should remain the primary route for ginsenoside administration in future studies, with the gastrointestinal tract being the most directly affected organ by ginsenosides [42]. However, there is currently no available research on the metabolism of ginsenosides in tumor patients. Therefore, future research should investigate whether there are any differences in the metabolism of ginsenosides between tumor patients and healthy individual (Tables 3, 4).

**Table 3:** Classification of anti-breast cancer effects of ginsenosides based on the basic hallmarks of tumors (2015–2023)

Ginsenoside type	Anti-breast cancer activity	Cell line	In Vivo/In Vitro	Type of tumor hallmarks	Target spot/signaling pathway	Refs.
Rg3	Activation of exogenous death pathway	MDA-MB-231, MDA-MB-453, BT-549	In Vivo/In Vitro	Resisting cell death	NF-κB p65, Bcl-2, Bax, caspase-3	[58]
20(S)-Rg3	Increase cell radiosensitivity and induce cell apoptosis	MDA-MB-231	In Vitro	Resisting cell death	Unknown	[59]
Rg5	Inducing apoptosis and autophagy death	MCF-7	In Vivo	Resisting cell death	PI3K/Akt pathway	[60]
Rg5	Caspase-dependent apoptosis can be induced by activating external death receptors and internal mitochondrial signaling pathways, and autophagy can be promoted	MCF-7	In Vitro	Resisting cell death	PI3K/Akt signaling pathway	[61]
Rk1	Activation of mitochondrial-mediated endogenous death pathway	MCF-7	In Vitro	Resisting cell death	PTEN/PI3K/Akt/mTOR signaling pathway	[62]
Rk1	Activation of mitochondrial-mediated endogenous death pathway	MDA-MB-231	In Vitro	Resisting cell death	ROS/PI3K/Akt pathway	[63]
Rg1	Increase cell DNA damage and induce cell apoptosis	MDA-MB-231	In Vitro	Resisting cell death	Akt, ERK, MAPK,ATM, H2AX, Rad51, TP53, BCl2, CDK2, NF-κB, STAT-3, MAPK, iNOS, MMP2, MMP9, TGFB1, VEGFA, EGFR, SOD, Catalase, GPx, GSH XRCC1, p21, TP53, apaf1, Bax, CASP3, CASP9, ROS, mitochondrial membrane potential	[64]
Rg1	Promote cell DNA damage, prevent the elevation of oxidative damage markers, restore antioxidant enzymes to near normal levels, inhibit the expression of cell proliferation and survival-related markers, regulate apoptosis markers, and downregulate invasion and angiogenesis markers	MB-MD-231	In Vitro	Resisting cell death, Tissue Invasion And Metastasis, Sustained Angiogenesis, Deregulating Cellular Energetics	TP53, Bcl-2, CDK2, NF-κB, STAT-3, MAPK, iNOS, MMP2, MMP9, TGFB1, VEGFA, EGFR, SOD, Catalase, GPx, GSH	[65]
Rh2	Upregulation of tumor suppressor gene expression, activation of extrinsic death pathway, and induction of apoptosis	MCF-7	In Vitro	Resisting cell death	caspase-9/p38 MAPK, p53, Bax, Bcl-2、PRAP	[66]
Rh2	Activation of the mitochondrial-mediated endogenous death pathway	MCF-7	In Vitro	Resisting cell death	Mitochondrial pathway	[67]
Rh4	Activation of extrinsic death pathways	MCF-7	In Vivo/In Vitro	Resisting cell death	Bcl-2, Bax, caspase-8, caspase-3, PARP	[68]
1f	Activation of the mitochondrial-mediated endogenous death pathway	MCF-7	In Vitro	Resisting cell death	Unknown	[69]
Rh1	Induction of apoptosis and autophagic death	MCF-7, HCC1428	In Vivo/In Vitro	Resisting cell death	ROS/PI3K/Akt pathway	[70]
Rh1	Induce mitochondrial dysfunction, activate mitochondria-mediated endogenous and exogenous death pathways	MDA-MB-231, BT549	In Vivo/In Vitro	Resisting cell death	PERK/eIF2α/ATF4/CHOP pathway, mtROS, caspase-3	[71]
Rh1	Activation of the mitochondrial-mediated endogenous death pathway	MD-MB-231	In Vitro	Resisting cell death, tissue invasion, and metastasis	STAT3/NF-κB pathway, MMP2, MMP9, ROS, VEGF-A	[72]
Rg2	Induction of autophagic cell death	MCF-7	In Vitro	Resisting cell death	p-p53, p-AMPK, p-ACC, Atg-7, LC3-II, p62	[75]
Rg2	Activation of the mitochondrial-mediated endogenous death pathway	MCF-7	In Vitro	Resisting cell death	AMPKpathway, mTOR	[76]
20(S)-PPT	Induction of apoptosis and nonprotective autophagy	MDA-MB-231, SUM-15-PT	In Vivo/In Vitro	Resisting cell death	Akt/mTOR signaling pathway	[77]
Rb1	Increase irreversible cell death under short infrared low light	MCF-7, 4T1	In Vitro	Resisting cell death	Unknown	[78]
Rb1	Induction of apoptosis	MCF-7	In Vitro	Resisting cell death	Unknown	[79]
Rh2	Inhibit cell proliferation, promote apoptosis, and cycle arrest	MCF-7, MD-MB-231	In Vivo/In Vitro	Self-sufficiency in growth signals	ERβ-TNFα pathway	[89]
Rg1	Exert estrogen regulation antagonism and inhibit cell proliferation	MCF-7	In Vivo/In Vitro	Self-sufficiency in growth signals	ER signalosome, EGFR, c-SrcER, cavolin-1	[90]
Rg6	It can change the paclitaxel resistance of cells by changing the chromosomal instability induced by stress hormones or steroid hormones, reduce the mitotic speed of cells, and inhibit cell proliferation	MCF-7, MDA-MB-468	In Vitro	Self-sufficiency in growth signals, Non-mutational epigenetic reprogramming	γ-tubulin, MTOC, GR, ER-α pathway	[91]
Rd	Eliminated VEGF-induced sprouting of blood vessels and inhibited the formation of blood vessels, and inhibited cell proliferation	MDA-MB-231	In Vivo/In Vitro	Sustained angiogenesis	Akt/mTOR/P70S6 pathway	[94]
Rg3	Inhibit proliferation, invasion, and angiogenesis, and enhance autophagy	MCF-7	In Vivo	Sustained angiogenesis, resisting cell death, tissue invasion and metastasis	EGFA, VEGFB, VEGFC, MMP2, MMP9, p62, mTOR, PI3K, Akt, JNK, Beclin-1, LC3-II/LC3-3 I	[95]
Rg3	Reduces cell stem-like properties and inhibits proliferation	MDA-MB-231, MCF-7	In Vitro	Unlocking phenotypic plasticity, tumor promotion inflammation, resisting cell death, tissue invasion, and metastasis	Akt-mediated self-renewal signaling	[101]
Rg3	Reduce the level of stemness markers of tumor cells, inhibit proliferation and metastasis, and promote apoptosis	MDA-MB-231, HCC1143	In Vivo/In Vitro	Unlocking phenotypic plasticity, tissue invasion and metastasis, resisting cell death	Akt/mTOR pathway, CD44, ALDH	[102]
Rh2	Inhibit the senescence phenotype and secretion phenotype of normal breast epithelial cells caused by doxorubicin treatment,	MCF-7, MCF-10A	In Vitro	Unlocking phenotypic plasticity, tumor promotion inflammation	NF-κB pathway, ROSj, SIRT 3, SIRT 5, SOD1, SOD2	[103]
Panaxatriol	It can change the phenotype of paclitaxel-resistant cells, inhibit the expression of inflammatory factors and stem cell-related genes, inhibit proliferation and invasion, and induce apoptosis	MDA-MB-231 PTX, SUM159-PR	In Vivo/In Vitro	Unlocking phenotypic plasticity, tumor promotion inflammation, resisting cell death, tissue invasion, and metastasis	IRAK1/NF-κB and ERK pathways	[49]
Rg3	Regulating promoter methylation of noncoding RNA inhibits cell proliferation and induces apoptosis	MCF-7	In Vitro	Non-mutational epigenetic reprogramming, resisting cell death, deregulating cellular energetics, tissue invasion and metastasis	lncRNA STXBP5-AS1, lncRNA RFX3-AS1, STXBP5, GRM1, RFX3, SLC1A1	[113]
Rg3	Regulating promoter methylation of noncoding RNA inhibits cell proliferation and induces apoptosis	MCF-7	In Vitro	Non-mutational epigenetic reprogramming, resisting cell death, deregulating cellular energetics	lnc RNA ATXN8OS, miR-424-5p, EYA1, DACH1, CHRM3	[114]
Rh2	Regulating promoter methylation of noncoding RNA inhibits cell proliferation and induces apoptosis	MCF-7	In Vitro	Non-mutational epigenetic reprogramming, resisting cell death, deregulating cellular energetics	ACOX2, FAM107A, lncRNA C3orf67-AS1	[115]
Rh2	Regulation of cell noncoding RNA levels inhibits cell proliferation	MCF-7, MDA-MB-231, T47D	In Vitro	Non-mutational epigenetic reprogramming, deregulating cellular energetics	lnc RNA CFAP20DC-AS1, BBX, TNFAIP3	[116]
Rg3	It inhibits tumor cell proliferation and promotes apoptosis by affecting gene methylation levels to affect the expression levels of tumor-associated proteins	MCF-7	In Vitro	Non-mutational epigenetic reprogramming, resisting cell death, deregulating cellular energetics	cell morphology-related pathway, TRMT1L, PSMC6, NOX4, ST3GAL4, RNLS, KDM5A	[117]
Rh2	It inhibits tumor cell proliferation and promotes apoptosis by affecting gene methylation levels to affect the expression levels of tumor-associated proteins	MCF-7	In Vitro	Non-mutational epigenetic reprogramming, resisting cell death, deregulating cellular energetics, avoiding immune destruction	CASP1, INSL5, OR52A1, CLINT1, ST3GAL4, C1orf198	[118]
Rh2	Regulating m6A methylation levels regulates the transcriptional activity and subcellular localization of oncogenic proteins	MDA-MB-157 , MCF-7	In Vitro	Non-mutational epigenetic reprogramming, deregulating cellular energetics	KIF26B, m6A RNA, ZC3H13/CBLL1	[119]
Rg1	Promoting mitotic defects in cells leads to delayed mitotic progression to inhibit cell proliferation	MDA-MB-231, MCF-7	In Vitro	Non-mutational epigenetic reprogramming, deregulating cellular energetics	Haspin, H3T3ph, Aurora B	[120]
CK	Reduce cellular glutamine utilization levels to inhibit proliferation and trigger apoptosis	MCF-7, BT474, MDA-MB-231, SUM159, HCC 1806	In Vivo/In Vitro	Deregulating cellular energetics, resisting cell death, deregulating cellular energetics	ATP, GLS1, GSH, ROS	[130]
Rg3	Inhibit glucose uptake by binding glucose transporters, reverse the level of immunosuppression in the tumor microenvironment, and reduce the levels of CAFs and collagen in the tumor microenvironment	4T1	In Vivo	Deregulating cellular energetics, avoiding immune destruction	Glut1, TGF-β/Smad pathway, CAFs, Collagen level, CD4, CD8, CD86, CD206, CD11b^+^/Gr-1^+^, CD4^+^FoxP3^+^, CD45	[131]
Rh2	Regulates the mitochondrial apoptotic pathway, inhibits glycolysis, and inhibits mitochondrial respiration	MCF-7	In Vitro	Deregulating cellular energetics, resisting cell death, deregulating cellular energetics	ROS, ATP, caspase-9, Bax, HK II	[132]
Rg3	Reverse drug resistance, rebuild TME, change macrophage phenotype, inhibit the expression of MDSC, AFs, and collagen fibers, improve tumor-associated inflammation, and promote tumor cell apoptosis	MCF-7	In Vivo/In Vitro	Avoiding immune destruction, Tumor promotion inflammation, resisting cell death	IL-6/STAT3/p-STAT3 pathway, MDSC, macrophages, TAFs, collagen fibers	[147]
Rg3	Reverses the level of immunosuppression in the tumor microenvironment and kills tumor cells by binding glucose-specific transporters	4T1	In Vivo	Avoiding immune destruction, tissue invasion, and metastasis	STAT3, CCL2, CD4, CD8, CD86, CD206, CD11b^+^/Gr-1^+^, CD4^+^FoxP3^+^, CD45, NF-κB, Bcl-2, Bax	[148]
Rg3	Reduce PD-1 expression of activated T cells and increase cytokine levels to promote T cell recognition and killing of breast cancer cells	MDA-MB-231 , BT-549	In Vivo/In Vitro	Avoiding immune destruction, resisting cell death	IFN-γ, IL-2, IL-9, IL-10, IL-22, IL-23, PD-1, PD-L1	[149]
Rh2	Improve tumor-associated inflammation, remodel the structure of TME, and reverse the immunosuppressive environment	4T1	In Vivo/In Vitro	Avoiding immune destruction, resisting cell death, Tumor promotion inflammation	α-SMA, TAFs, CD31, CD11b^+^/F4/80^+^/CD86^+^, M1 macrophage, M2 macrophage, IL-6, CD4, CD8	[150]
Rg3	Inhibit angiogenesis and cell invasion, enhance autophagy, and inhibit cell proliferation	MCF-7	In Vitro	Tissue invasion and metastasis, sustained angiogenesis, resisting cell death, deregulating cellular energetics	VEGFA, VEGFB, VEGFC, MMP2, MMP9, p62, mTOR, PI3K, Akt, JNK, Beclin-1, LC3-II/LC3-I	[156]
Rh2	Reduce cell invasion-related proteins to inhibit cell invasion	MDA-MB-231, MCF-7	In Vitro	Tissue invasion and metastasis	Anxa2-K301A, NF-κB, E-cadherin, N-cadherin, Snail1, Twist, Slug, SIP1, MMP-2, MMP-9, Myc	[157]
Rd	Changing the expression level of non-coding RNA inhibits cell invasion	4T1	In Vivo/In Vitro	Tissue invasion and metastasis	microRNA-18a, Smad2	[158]
CK	Inhibit cell proliferation and invasion, induce apoptosis	MCF-7	In Vitro	Tissue invasion and metastasis, resisting cell death	N-cadherin, vimentin, FN, E-cadherin, PI3K/Akt pathway	[159]
20(S)-PPD	Reducing the expression level of epidermal growth factor inhibits cell proliferation, metastasis, invasion, and metastasis	MDA-MB-231 , SUM159	In Vivo/In Vitro	Tissue invasion and metastasis, resisting cell death, deregulating cellular energetics	ERK1/2, p38, JNK pathway, EGFR-mediated MAPK pathway	[160]

**Table 4:** Related effects of ginsenoside in attenuating adverse reactions induced by chemical antitumor drugs

Ginsenoside type	Anti-breast cancer activity	Cell line	In vivo/in vitro	Drugs that cause adverse reactions	Signaling pathway/target spot	Refs.
Rg2	Reducing cardiomyocyte apoptosis induced by trastuzumab therapy	/	In Vivo	Trastuzumab	caspase-3, caspase-9, Bax	[167]
Rg2	Promote the protective autophagy of myocardial cells and avoid the apoptosis of myocardial cells caused by trastuzumab therapy	Human primary HCMs	In Vitro	Trastuzumab	p-Akt, p-mTOR, beclin 1, LC3, ATG5	[168]
Rh2	Cardiotoxicity is reduced by inhibiting cardiac histopathological changes, apoptosis and necrosis, and consequent inflammation. Pathological remodeling is attenuated by reducing fibroblast to myofibroblast transformation (FMT) and endothelial-mesenchymal transformation (EndMT). It can promote the senescence of myofibroblasts and reverse the differentiation of myofibroblasts established in EndMT to alleviate fibrosis	MDA-MB-231, HUVEC	In Vivo	Doxorubicin	caspase-3, caspase-7, caspase-9, TNF-α, IL-6, IL-1β, CD31, CD206, fα-SMA, Vimentin, Smad2, Smad3	[169]
Rg1	Promote that specific combination of doxorubicin and tumor cells and avoid the apoptosis of myocardial cells induced by doxorubicin	/	In Vivo	Doxorubicin	ROS, p53, caspase-3	[170]
Panaxatriol	Reversing paclitaxel resistance	MDA-MB-231, SUM159	In Vivo	Paclitaxel	RAK1/NF-κB and ERK signaling pathways, S100A7/9, inflammatory factors (IL6, IL8, CXCL1, CCL2), cancer stem cell-related (OCT4, SOX2, NANOG, ALDH1, CD44)	[49]

At present, there is no research on the metabolism of ginsenoside in tumor patients. Therefore, it is speculated that ginsenoside is also metabolized in human body through liver-kidney pathway. In the clinical study with only a few ginsenoside, the relationship between the dosage of ginsenoside and adverse reactions was reported [43–45]. In vivo studies of healthy volunteers found that ginsenoside at therapeutic dose (1–2g/day) would not cause serious adverse reactions. After taking ginsenoside orally to healthy volunteers, the reported side effects are mainly mild, mainly dizziness, insomnia, nervousness and uterine bleeding. However, the study found that the adverse reactions, such as dizziness, insomnia, nervousness and uterine bleeding, would disappear after one month of treatment. The most common adverse reactions after ginsenoside administration for one month are constipation, dyspepsia, insomnia and hot flashes, and these adverse reactions are mild. Therefore, it can be concluded that ginsenoside will not cause serious treatment-related adverse reactions when it is treated in vivo at therapeutic dose. This also means that ginsenoside is safe and tolerable in the treatment of breast cancer patients.

## Mechanism of action of ginsenosides altering breast cancer hallmarks

**Fig. 4:**
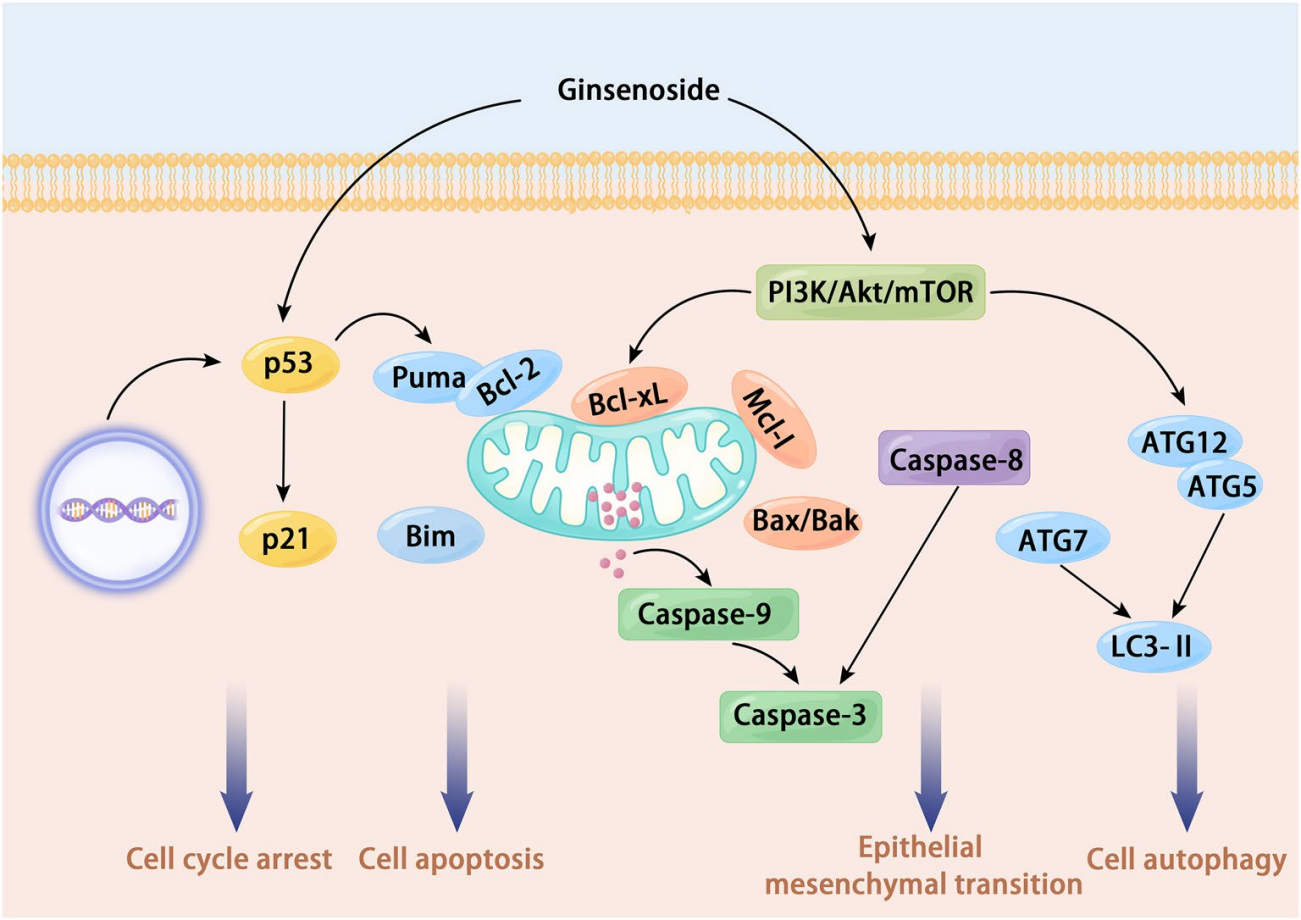
The main molecular mechanism of ginsenosides anti-breast cancer. Ginsenosides can treat breast cancer through various targets and multiple signaling pathways. Cell cycle arrest, cell apoptosis, EMT, and cell autophagy are the most common phenotypic changes after ginsenoside intervention in breast cancer cells, and these phenotypic changes can achieve the purpose of treating breast cancer

Regardless of the stereotype, ginsenosides have been studied in several in vivo and in vitro models of breast cancer, and various mechanisms of the therapeutic effect of ginsenosides have been revealed. The mechanisms of action of ginsenosides are achieved by altering the characteristics of breast cancer cells (Fig. 4). These mechanisms include induction of apoptosis, inhibition of cell proliferation, induction of autophagy through upregulation of autophagy-related molecules, inhibition of metastasis and angiogenesis, cell cycle arrest, immunomodulatory effects, induction of cellular phenotypic changes, prompting cellular epigenetic reprogramming, remodeling of cellular abnormal energy metabolism, and suppression of tumor cell-induced inflammation.

Although most currently discovered ginsenosides have been identified as naturally occurring compounds or compounds that can be metabolically produced, some can be chemically synthesized [46]. The current generation of ginsenosides with definite anti-breast cancer activity has become a major research hotspot, especially Rg3 (12 studies), Rh2 (11 studies), Rg1 (5 studies), and Rg2 (4 studies). Compound K is the in vivo metabolite of natural ginsenosides, and many ginsenosides undergo metabolism in the body to eventually form compound K. In Asia, boiling is one of the most common methods for processing natural Radix Ginseng. It has been found that Radix Ginseng, processed by steaming, decreases the major ginsenosides Rb1, Rb2, Rb3, Rc, Rd, Re, and Rg1 and increases the specific ginsenosides Rg2, Rg3, Rg5, Rh1, Rh2, and Rh4, leading to an increase in the ginsenoside components in Radix Ginseng that have anti-breast cancer activity [47]. The less abundant ginsenosides, such as 20(S)- and 20(R)-ginsenosides, are scarce in natural Radix Ginseng but are equally cytotoxic to breast cancer cells. The structural differences between 20(S)-Rg3 and 20(R)-Rg3 are attributed to the position of the C-20 hydroxyl group. Studies have indicated that 20(S)-Rg3 exhibits superior inhibitory activity against breast cancer compared to 20(R)-Rg3 [48]. Most anti-breast cancer studies involving ginsenosides have focused on in vitro assays using breast cancer cell lines such as MCF-7 (luminal type A) and MDA-MB-231 (triple-negative breast cancer). However, there are limited in vivo studies available. Some studies have explored the potential of ginsenosides in reversing drug resistance in breast cancer cell lines specifically resistant to certain chemotherapeutic agents [49]. Clinical studies have shown that Ginsenoside Rg3 treatment could enhance the anti-tumor efficacy of chemotherapy agents (such as Capecitabine, Docetaxel, and Cisplatin) in patients with advanced breast cancer [50–52]. Additionally, ginsenoside therapy has been found to reduce the adverse effects caused by chemotherapy, improve immune function, and enhance patient quality of life [53]. However, it is important to note that current studies on ginsenoside therapy for breast cancer are predominantly conducted in vitro, with only a limited number of in vivo models being explored. The following mechanisms associated with ginsenosides in breast cancer are discussed below.

### Ginsenosides can induce apoptosis in breast cancer cells

Resisting cell death is one of the fundamental characteristics of tumor cells. Cells that experience irreversible DNA damage undergo programmed cell death, also known as apoptosis [54]. However, tumor cells have developed mechanisms to evade this program, making them apoptosis-resistant. Apoptosis is a fundamental type of programmed cell death and plays a crucial role in the mechanism of action of various anti-tumor drugs. Apoptosis occurs mainly through the extrinsic death receptor pathway and the intrinsic mitochondrial pathway [55]. These pathways lead to the activation of cysteine proteases, which can cleave to generate different substrates and then cause cell death [56]. Promoting breast cancer cell pyroptosis by extrinsic death receptor pathway to induce cellular cysteine proteases activation is one of the main and most directly effective mechanisms of ginsenosides anti-breast cancer. Several studies have investigated the effects of different ginsenosides on breast cancer cells, and they have identified several ginsenosides, such as Rh2, Rh4, Rk1, Rg3, and Rg5, that are capable of inducing apoptosis in various subtypes of breast cancer cells, including MCF-7 and MDA-MB-231 cells.

Ginsenoside Rg3 is currently recognized as one of the most promising agents for tumor treatment among the various ginsenosides. Numerous basic and clinical studies have demonstrated its therapeutic efficacy against various tumors [57]. Moreover, several studies have confirmed that ginsenoside Rg3 exerts therapeutic effects against breast cancer through multiple targets and mechanisms. In laboratory studies, both in vivo and in vitro experiments have consistently shown the ability of ginsenoside Rg3 to induce apoptosis in breast cancer cells, highlighting its potential as an effective treatment option for breast cancer. After ginsenoside Rg3 intervention in breast cancer cells [58], cell proliferation viability was inhibited, and apoptotic cells with DNA fragmentation cells increased. For ginsenoside-induced apoptosis in triple-negative breast cancer cells, the mechanism is mainly through inhibiting NF-κB activation, decreasing NF-κB p65 and Bcl-2 protein expression, and increasing the ratio of Bax and caspase-3 protein expression to Bax/Bcl-2 to achieve therapeutic efficacy. The elucidation of the mechanism of ginsenoside Rg3 in breast cancer treatment has contributed to its success in clinical trials as a potential breast cancer drug. Interestingly, the intervention of 20(S)-Rg3 has been shown to increase the sensitivity of MDA-MB-231 cells to radiotherapy. However, the precise mechanism behind this effect remains unclear [59]. Ginsenoside Rg5 is also an active component that can induce breast cancer cells apoptosis in various ways. Ginsenoside Rg5 [60, 61] and ginsenoside Rk1 [62, 63] have shown promising anti-tumor effects on MCF-7 cells by binding to active domains of PI3K proteins and increasing the levels of reactive oxygen species expression to inhibit PI3K/Akt signaling and via the extrinsic apoptotic pathway, intrinsic apoptotic pathway, and autophagic pathway. Other ginsenosides can also induce apoptosis in breast cancer cells through multiple pathways. For example, Rg1 [64, 65] can induce apoptosis by enhancing the expression levels of apoptotic genes and altering mitochondrial membrane potential, Rh2 [66, 67] composites induce apoptosis by activating the caspase-9/p38 MAPK signaling pathway and mitochondrial pathway, and Rh4 [68] induces apoptosis by blocking the cell cycle and upregulating the expression levels of apoptotic proteins. The above findings demonstrate that despite their heterogeneous chemical structures, different ginsenosides can induce apoptosis in breast cancer cells, offering potential treatment options. Additional ginsenosides, such as 1f [69] and Rh1 [70–72], have been found to induce apoptosis in breast cancer cells by affecting multiple signaling pathways associated with both the intrinsic and extrinsic pathways.

Cell autophagy is a cellular process where damaged proteins or organelles are enclosed within double-membrane autophagic vesicles, which are then delivered to lysosomes for degradation and recycling [73]. Traditionally, autophagy and apoptosis have been regarded as mutually inhibitory processes. However, research has shown that certain pharmacological interventions can simultaneously activate both autophagy and apoptosis, synergistically promoting cell death [74]. It has been observed that some ginsenosides can induce irreversible autophagy and apoptosis in cells, leading to therapeutic effects against breast cancer. Ginsenoside Rg5 [60, 61] enhanced the expression and activation of autophagy-related and apoptotic proteins to promote the apoptosis of MCF-7 cell xenografts by suppressing the activation level of the PI3K/Akt signaling pathway in in vivo experiments. Treatment of MCF-7 cells with ginsenoside Rg2 [75] at the in vitro level can lead to upregulation of p-p53, p-AMPK, p-ACC, Atg-7, and LC3-II levels and reduction of p62 levels in a concentration-dependent manner to promote autophagy and induce apoptosis. Likewise, intervention with Rg2 [76] was found to sensitize breast cancer cells to autophagic death and apoptosis. The application of (20S)-PPT [77] in vivo and in vitro can enhance the expression levels of autophagy-related proteins, autophagosomes, and apoptotic protein families (Bcl-2, Bax) by inhibiting the expression level of Akt/mTOR pathway, and achieve the dual effects of autophagy induction and apoptosis on the premise of ensuring the safety of the drugs. The nanoparticle carriers of Rb1 [78] and Rb1 [79] all showed cytotoxicity to breast cancer cells in vitro, although it remains unclear how they induce apoptosis.

### Ginsenosides alter self-sufficient growth signaling in breast cancer cells

It is well-established that normal mammary epithelial cells require the activation of mitogenic growth signals to progress from a quiescent to an active proliferative state [80]. However, breast cancer cells do not require mitogenic growth signals to continuously maintain an active proliferative state [81]. These signals are transmitted into the cell through transmembrane receptors, which combine different types of signaling molecules: diffusible growth factors, extracellular matrix components, and intercellular adhesion/interaction molecules [82]. Without this stimulatory signal, normal mammary epithelial cells of any type fail to proliferate. In contrast, many oncogenes in breast cancer cells mimic normal growth signals in various ways to stimulate the proliferation of tumor cells [83].

Although most soluble mitogenic growth factors are synthesized and secreted by one cell type to stimulate the proliferation of another, tumor cells can produce mitogenic growth factors to eliminate the reliance on mitogenic growth factors for other cells within the tissue [84]. In approximately 25% of patients with tumors, Ras proteins exist in structurally altered forms that allow them to release numerous mitogenic signals into the cell without sustained stimulation by their normal upstream regulators [85]. An example of the interplay between signaling pathways in promoting cell proliferation involves the direct interaction of Ras proteins with PI3K, leading to the simultaneous activation of growth signals and survival signals within the cell [86]. This phenomenon is driven by the persistent activation of oncogenes and the inactivation of tumor suppressor genes. In estrogen receptor-positive breast cancer cells, the primary reliance for proliferation is on estrogen receptor signaling, which activates growth factor signaling [87]. When estrogen expression and signaling are inhibited, the intracellular growth signal is blocked, ultimately inducing apoptosis [88]. Several recent studies have shown that ginsenoside intervention can inhibit the sustained proliferation of breast cancer cells by inhibiting estrogen receptors and their signaling.

Ginsenoside Rh2 has been studied in MCF-7 cells and shown to reduce the protein level of estrogen receptor alpha (ERα) while increasing the mRNA and protein levels of ER-beta (ER-β) and tumor necrosis factor-alpha (TNF-α). Through the β-TNF-α signaling pathway, ginsenoside Rh2 induces apoptosis and G1/S arrest, leading to anti-tumor effects in xenograft mice [89]. On the other hand, ginsenoside Rg1 has been found to induce the translocation of ER to the plasma membrane through caveolin-1 in MCF-7 cells, leading to the formation of a signaling complex and phosphorylation of extracellular signal-regulated protein kinase (ERK) and mitogen-activated protein kinase (MAPK). Additionally, it activates epidermal growth factor receptor and cellular nonreceptor tyrosine kinase independently of ER, exerting estrogenic effects by rapidly activating membrane-associated ER and G protein-coupled estrogen receptors. This, in turn, inhibits the activation of proliferative signals in breast cancer cells [90]. Another study found [91] that intervention of Rg6 could block cortisol/cortisone-induced deregulation of microtubule organizing centers as well as ER signaling in breast cancer cells, alter stress hormone or steroid hormone-induced chromosomal instability, and inhibit aberrant activation of tumor cell proliferative signaling. The stress hormone cortisol and cortisone have been shown to elevate the expression of γ-tubulin, increasing the number of microtubule organizing centers and promoting resistance to paclitaxel in breast cancer cells. However, the intervention of ginsenoside Rg6 has been found to block the dysregulation of MTOCs induced by cortisol/cortisone and inhibit ER-alpha signaling.

### Ginsenosides block breast cancer angiogenesis

Targeting angiogenesis inhibition has emerged as a promising therapeutic approach for treating breast cancer, yielding favorable results across different subtypes. Cancer growth and metastasis rate can be controlled by inhibiting the formation of new blood vessels in breast tumors. Additionally, blocking the supply of oxygen and nutrients necessary for breast cancer cell proliferation can induce apoptosis or autophagy in these cells [92]. Although commonly used anti-angiogenic drugs in clinical practice (such as Bevacizumab, Apatinib, Anlotinib, etc.) can effectively inhibit neoangiogenesis in breast cancer, they can also lead to adverse effects such as hypertension, thrombosis, and hemorrhage, thereby impacting patients' quality of life [93]. However, certain ginsenosides have shown potential as inhibitors of breast tumor angiogenesis, offering a more targeted and potentially safer approach to breast cancer treatment.

Ginsenoside Rg1 [65] exhibited significant anti-breast cancer activity in in vitro experiments by promoting tumor cell DNA damage and undergoing apoptosis through alteration of mitochondrial membrane potential and ROS levels, and in vivo experiments by downregulating angiogenesis, invasion, and EMT markers mediated by anti-oxidant enzymes. Ginsenoside Rd [94] could dependently inhibit vascular endothelial growth factor-induced migration, tube formation, and proliferation of primary cultured human umbilical vascular endothelial cells, and the activation of Akt/mammalian target of the rapamycin signaling cascade in VEGF-induced human umbilical vascular endothelial cells was inhibited under normoxic or hypoxic conditions to abolish aortic ring vessel sprouting and vessel formation. In an intraperitoneal xenograft mouse model constructed with MDA-MB-231 cells, Rd significantly reduced the volume and weight of breast cancer tumor tissues, decreased tumor angiogenesis, and inhibited breast cancer cell proliferation by suppressing Akt/mTOR/p70S6 kinase signaling in a dose-dependent manner. Rg3 [95] yielded a strong inhibitory effect on tumor growth in MDA-MB-231 cells tumor-bearing mice when combined with recombinant human endostatin, as well as inhibiting angiogenesis and cell invasion and enhancing cell autophagy.

### Ginsenosides can transform breast cancer cell phenotypes

In normal tissues, the transformation of progenitor cells into mature tissue cells allows them to assume specialized functions and achieve homeostasis by ceasing their growth. This process is facilitated through development and terminal differentiation, typically resulting in an anti-proliferative state. However, in the context of breast carcinogenesis, the capacity for phenotypic plasticity becomes deregulated, allowing breast cancer cells to evade or escape from the terminally differentiated state. This abnormal phenotypic plasticity is a key factor in the development of breast cancer [96]. Breast cancer cells, which originate from normal cells, may undergo a reversal of their differentiation state, dedifferentiating back to a progenitor-like state as a potential therapeutic strategy [97]. Conversely, breast cancer cells arising from progenitor cells programmed to follow a pathway of end-stage differentiation may disrupt this process and maintain themselves in a partially differentiated, progenitor-like state. Some breast cancer cells may differentiate into fully formed tumor cells, referred to as breast cancer stem-like cells, while others retain characteristics of progenitor cells [98]. Breast cancer stem-like cells are more proliferative, invasive, and metastatic than normal breast cancer cells [99]. Therefore, inducing breast cancer cells to differentiate into a progenitor-like state or inducing breast cancer stem-like cells into normal breast cancer cells by drug or gene therapy may be an unusual approach to treating breast cancer.

Inducing differentiation in tumor cells can modulate the expression levels of selective regulators involved in cellular plasticity and signal transduction, such as Wnt signaling, Hippo signaling, Notch signaling, and Hedgehog signaling. This modulation ultimately affects the cellular phenotype [98]. Altering phenotypic plasticity in tumor cells can be achieved through three main subclasses: dedifferentiation of mature cells to progenitor states, blocking differentiation to maintain cells in a progenitor/stem cell state, and transdifferentiation to alternative cell lineages [100]. Inducing differentiation has significant implications for breast cancer formation, malignant progression, and response to therapy and appears effective across different breast cancer types [100]. While gene therapy is currently limited in clinical applications due to ethical considerations, drug-induced differentiation is an attractive direction in the research of induced differentiation of tumor stem-like cells. Some ginsenosides have shown the ability to modulate various regulators of cell plasticity and signal transduction, leading to the induction of differentiation in breast cancer stem-like cells. These ginsenosides can transform breast cancer stem cells into normal breast cancer cells, exerting cytotoxic effects and contributing to breast cancer treatment.

Ginsenoside Rg3 [101] has been shown to reduce the clonogenic capacity of breast cancer stem-like cells by decreasing the expression levels of CD44, a surface marker of breast cancer stem-like cells, to induce their differentiation through Akt-mediated self-renewal signaling to decrease the expression and localization of SOX2, BMI-1, and hypoxia-inducible factor-1α. The combination of C3 [102], a distinct isoform of Rg3, with Rg3, can convert CD44^+^ MD-MB-231 cells into normal CD44^−^ MD-MB-231 cells and significantly inhibit cell migration ability. C3 alone also achieves therapeutic effects on breast cancer by binding to IGF-1R, Akt, and mTOR, reducing the primary focus and metastatic load in tumor-bearing mice. Rh2 [103] decreased the protein expression levels of TRAF6, p62, and phosphorylated IKK and IKB and inactivated NF-κB activity by inhibiting IL-8 secretion mediated by regulation of ROS levels and mitochondrial autophagy. Ginsenoside panaxatriol [49] induces the transition of breast cancer stem cells into normal breast cancer cells by repressing paclitaxel-resistant breast cancer stem cell-associated genes (OCT4, SOX2, NANOG, CD44, ALDH1) and by inhibiting IRAK1/NF-κB and ERK pathway reduced inflammatory cytokines (IL-6, IL-8, CXCL1, CCL2) expression, which contributed to resensitization of paclitaxel-resistant cells to paclitaxel.

### Ginsenosides can reverse nonmutational epigenetic reprogramming in breast cancer cells

Genome instability and mutation are fundamental in breast cancer pathogenesis [104]. There is an increasing consensus that cancer cells can regulate gene expression in various ways, such as regulating noncoding RNAs, altering chromatin states, and affecting epigenetic modifications [105]. It is well-established that the genomes (DNA of normal individuals and breast cancer patients are identical. In normal adults, the formation of long-term memory relies on various mechanisms, including modifications to genes and histones, changes in chromatin structure, and the activation or repression of specific genes through intricate feedback loops. However, in breast cancer patients, these regulatory switches are dysregulated, leading to the activation of numerous oncoproteins and the suppression of tumor suppressor proteins, ultimately contributing to the initiation and progression of breast cancer. This model involves alterations in gene expression that are controlled purely through epigenetic mechanisms, a phenomenon referred to as “nonmutational epigenetic reprogramming” [106]. Nonmutational epigenetic reprogramming mainly includes chromatin remodeling complexes, histone modifications, non-coding RNAs, and other epigenetic mechanisms. The nonmutational epigenetic reprogramming of breast cancer cells is not restricted to breast cancer cells themselves but comprises three important aspects: epigenetic regulation of stromal cell types in the breast cancer cell microenvironment, epigenetic regulation of breast cancer cell heterogeneity, and epigenetic regulation of the breast cancer cell microenvironment [107].

Advanced research techniques have enabled a better understanding of breast cancer epigenomic heterogeneity. These techniques include profiling genomewide DNA methylation, histone modification, chromatin accessibility, posttranscriptional modification, and translation of RNA [108]. These technologies provide us with a better understanding of how nonmutational epigenetic reprogramming plays a role in the development of breast cancer. For example, non-coding RNAs can overexpress or degrade target proteins; chromatin remodeling leads to changes in the structural position of nucleosomes affecting transcriptional regulation of genes, and modifications of histones through acetylation, methylation, phosphorylation, ubiquitination, ADP-ribosylation of histones [109]. Nonmutational epigenetic reprogramming ultimately suppresses tumor suppressor protein expression and (or) the activation of oncoprotein expression [110]. Interventions targeting nonmutational epigenetic inheritance have shown promise for breast cancer treatment, with two main approaches being gene therapy and drug therapy [111]. While gene therapy faces limitations due to ethical considerations in clinical applications, pharmacotherapy aimed at regulating breast cancer through nonmutational epigenetic reprogramming has attracted significant interest. Importantly, modulating the expression of oncoproteins and/or tumor suppressor proteins through chromatin remodeling complexes, histone modifications, non-coding RNAs, and other mechanisms can suppress tumor growth, invasion, and metastasis [112]. Breast cancer epigenetic drugs currently under investigation include DNA methyltransferase inhibitors, histone methyltransferase inhibitors, histone demethylase inhibitors, histone deacetylase inhibitors, bromodomain, and extra-terminal, isocitrate dehydrogenase inhibitors. However, studies on ginsenoside treatment for breast cancer have revealed its potential to intervene in breast cancer cells through various nonmutational epigenetic mechanisms, including chromatin remodeling complexes, histone modifications, and non-coding RNAs.

Ginsenosides Rg3 [113, 114] and Rh2 [115, 116] have been found to regulate the expression levels of breast cancer-related genes and proteins through the involvement of various non-coding RNAs. For example, they can modulate the expression of genes such as BBX, TNFAIP3, and SLC1A1 through non-coding RNAs like lnc STXBP5-AS1, lnc RFX3-AS1, miR-3614-3p, and others, thereby exerting anti-tumor activity. Additionally, Rg3 [117] and Rh2 [118, 119] have been shown to regulate the methylation levels and demethylation levels of breast cancer-related genes and proteins, including TRMT1l, KDM5A, and CAS1, through mechanisms such as N6-adenylate methylation, thus altering the breast cancer-associated microenvironment, enhancing immunogenicity, and inhibiting cancer cell growth. Furthermore, Rg1 [120] has been demonstrated to inhibit the phosphorylation of histone H3Thr3 mediated by Haspin kinase, leading to an increase in the width of the metaphase plate and spindle instability during cancer cell division, resulting in a delay in the progression of mitotic differentiation and inhibition of proliferation in breast cancer cells. Similarly, intervention with Rg6 [91] can induce chromosomal instability, which resensitizes paclitaxel-resistant breast cancer cells to paclitaxel treatment.

### Ginsenosides drive remodeling of energy metabolism reprogramming in breast cancer cells

In recent years, many reports have indicated a close relationship between breast cancer-related cancer signaling pathways and energy metabolism activities. Breast tumor cells undergo metabolic reprogramming during tumorigenesis, including enhanced glycolysis, the tricarboxylic acid cycle, glutaminolysis, and fatty acid biosynthetic processes [121]. It is important to note that the specific metabolic changes can vary between different subtypes of breast cancer cells. However, this metabolic reprogramming is considered a crucial characteristic of breast cancer occurrence and progression. The continuous proliferation and invasive behavior of breast cancer cells necessitate a substantial energy supply to sustain their activities. In normal breast cancer cells with sufficient oxygen, glucose undergoes oxidative phosphorylation in the cytoplasm to produce carbon dioxide and energy via glycolysis to produce pyruvate, followed by oxidative phosphorylation in the mitochondria. However, when oxygen is insufficient, glucose generates lactate and energy in mitochondria undergoing anaerobic glycolysis [122]. Otto Warburg first observed the abnormal energy metabolism of cancer cells, noting that tumor cells can take up glucose in large quantities and undergo glycolysis even under sufficient oxygen conditions [123]. Recent studies have found that tumor cells increase the uptake of glucose and energy substrates, such as amino acids and fat, in large amounts to meet the energy demand of rapid tumor cell proliferation [124]. Oncogenes associated with breast cancer promote increased uptake of energy substances mainly by promoting breast cancer cell proliferation, inducing cell transfer, and reducing apoptosis. Furthermore, breast cancer cells can take up certain metabolites, such as lactate, and serve as signaling molecules that promote proliferation and invasion [125].

The reprogramming of energy metabolism is largely controlled by key proteins involved in energy metabolism that participate in programming cancer's fundamental hallmarks [126]. Targeted inhibition of these key proteins could achieve anti-tumor effects by regulating the mode of energy metabolism reprogramming. Over the past decade, targeting tumor energy metabolism has emerged as a new hotspot in developing novel anti-tumor drugs. Tumor-associated alterations in bioenergetic metabolism include aerobic glycolysis and tricarboxylic acid cycle, mitochondrial respiration, glutamine metabolism, and so on. These important metabolic processes can serve as potential anti-tumor drug targets [127]. However, there is significant heterogeneity in metabolic reprogramming among different tumor cells, emphasizing the need to develop drugs according to the metabolic reprogramming characteristics of different tumor cells. For instance, the anti-diabetic drug metformin is currently being evaluated for its potential anti-tumor effects in breast cancer patients. Metformin can interfere with mitochondrial complex I action, thus enabling AMPK activation. In parallel, the inhibitory effect of metformin on mitochondrial respiration has the potential to contribute to its anti-tumor effects [128]. Of note, the mechanism by which metformin reduces cancer incidence and mortality does not appear to be related to its pharmacological effects on glycemia. It is widely thought that its anti-tumor effects are mainly mediated through inhibiting AMPK-dependent and independent hepatic gluconeogenesis [129]. Ginsenosides can also contribute to remodeling energy metabolism reprogramming in breast cancer cells by regulating key energy metabolism proteins.

It has been shown that ginsenoside CK [130], when administered to TNBC cells, inhibits glutamine consumption and glutamate production by downregulating the expression of glutaminase 1. This leads to reduced cellular ATP production and the utilization of amino acids involved in glutamine metabolism. Consequently, TNBC cells experience glutathione depletion and accumulation of reactive oxygen species, leading to the regulation of apoptosis-related protein expression levels and induction of TNBC cell apoptosis. Rg3 and DTX synthesized DTX-loaded Rg3 liposomes (Rg3-Lp/DTX) [131] exhibit enhanced accumulation at the tumor site due to the interaction between the glycosyl moiety of Rg3 exposed on the liposome surface and the overexpressed glucose transporter 1 on breast cancer cells. This allows Rg3-Lp/DTX to accumulate at the tumor site and block glucose transport by glucose transporter 1. Additionally, it reverses activated cancer-associated fibroblasts (CAFs) to a resting phase and attenuates the dense stromal barrier by inhibiting the secretion of TGF in tumor cells and modulating TGF/Smad signaling. As a result, it achieves a dual effect of modulating the tumor microenvironment and enhancing the anti-tumor effect of DTX. 2-Deoxy-Rh2 [132] exhibits dual anti-tumor activities by inhibiting glycolysis and mitochondrial respiration. It achieves this by binding to the active site of hexokinase II, thereby reducing the activation of anaerobic glycolysis compared to mitochondrial respiration. Furthermore, it regulates the mitochondrial apoptotic pathway.

### Ginsenosides prevent breast cancer cells from evading immunogenic cell death

The immune system constantly monitors normal cells and tissues and is responsible for the recognition and elimination of the vast majority of potential cancer cells and early tumors [133]. However, when immune surveillance functions are impaired, cancer cells can evade detection, and the immune system may mistakenly perceive tumor tissue as normal, allowing the uncontrolled proliferation of tumor cells [134]. Studies using carcinogens to induce tumor development in mice have shown that immunodeficient mice develop tumors more frequently and at a faster rate than mice with intact immune activity [135]. Similar results have been observed in experiments involving transplanting human tumor cells into animals. Clinical research also supports the presence of anti-tumor immune responses in human cancers. For instance, immune cell infiltration, including natural killer cells, can be observed in the tumor tissues of colon and ovarian cancer patients [136]. Patients with a higher degree of immune cell infiltration, known as “hot” tumors, tend to have a better prognosis compared to tumors with limited immune cell infiltration, referred to as “cold” tumors [137]. Various immune cells are present in the tumor microenvironment, including tumor-infiltrating lymphocytes, macrophages, and neutrophils [138]. Among these, CD8^+^ T cells play a critical role in targeted killing of tumor cells. Upon activation, CD8^+^ T cells release intracellular granule toxins that induce tumor cell death [139]. The progression of tumors is viewed immunologically as consisting of three phases: tumor elimination, tumor equilibrium, and tumor escape [140]. During the tumor escape phase, tumor cells employ mechanisms to evade recognition and killing by secreting immunosuppressive factors and converting T cells into immunosuppressive T cells, such as exhausted T cells and anergic T cells that do not have tumor recognition or killing functions [141]. Additionally, tumor cells may recruit immunosuppressive inflammatory cells, such as regulatory T cells and myeloid-derived suppressor cells, which inhibit the action of cytotoxic lymphocytes targeting tumor cells [142].

Approaches to treating tumors by targeting tumor immune escape-related mechanisms have been demonstrated in laboratory studies and clinical studies. The methods of tumor immunotherapy can mainly be divided into two main categories, immune cell therapy and cytokine therapy [143]. Immune cell therapy involves increasing the number of immune cells or modifying the antigen presentation of immune cells to enhance their recognition and induction of immunogenic cell death in tumor cells [144]. However, the complexity of the preparation process, high cost, and potential side effects limit the widespread use of immune cell therapy in clinical settings. Conversely, cytokine therapy involves using cytokines to block the secretion of immunosuppressive factors by cancer cells, allowing immune cells to exert their normal immunogenic cell death effects on tumor cells [145]. Cytokine therapy is currently the most commonly used immunotherapy in the clinic. The expression of these immunosuppressive factors by cancer cells can be blocked by monoclonal antibodies against proteins such as PD-1, PD-L1, and CTAL-4, which prompt normal recognition by immune cells to kill cancer cells [146]. Breast cancer cells often possess robust and complex immune escape mechanisms that hinder the normal recognition and elimination of cancer cells by immune cells. However, certain natural products, including ginsenosides, have been shown to enhance immunogenic cell death of breast cancer cells by inhibiting immune factors secreted by cancer cells, modifying antigen presentation by immune cells, and increasing immune cell infiltration.

Modifying ginsenoside Rg3 into Rg3 liposomes [147] can regulate the expression levels of immunosuppressive factors such as TGF-β and IL-6, as well as related signaling pathways. This leads to reshaping the expression levels of immune cells and fibroblasts in the immune microenvironment, improving the immunosuppression observed in breast cancer tissues, and enhancing immune cells' recognition and killing function against breast cancer cells. Rg3 liposomes, combined with chemotherapeutic agents [148], can inhibit breast cancer proliferation and invasion by binding to specific sites on breast cancer cells and altering the components of the tumor microenvironment. Rb3 incorporated into carbon nanotubes [149] can impact the PD-1/PD-L1 axis in a co-culture system of T cells and triple-negative breast cancer cells. This reduces the expression of T cell-related inhibitors, promotes the recognition and killing of T cells targeting breast cancer cells, and achieves an anti-cancer effect. Rh2 [118] can increase immunogenic cell death and inhibit the growth of breast cancer cells by modifying the epigenetic methylation of genes involved in immune response and tumorigenesis. Multifunctional liposomes containing Rh2 [150] can easily interact with tumor cells through glucose transporters (GLUT), effectively inhibiting the growth of breast cancer by reshaping the cellular composition of the tumor microenvironment and reversing the immunosuppressive environment.

### Ginsenosides block breast cancer cell invasion and metastasis

In general, most cells in the body have a fixed spatial localization within the tissues they belong to and cannot detach and survive elsewhere in the body. However, during the early stages of tumor development, tumor cells proliferate uncontrollably in the primary tumor site and disrupt the organ's normal functions. The majority of cancer-related deaths, more than 90%, are attributed to multi-organ failure caused by the development of metastatic lesions in other organs [151]. Current research suggests that cancer cells when they detach from the primary tumor site and undergo invasion and metastasis, undergo a process called “epithelial-mesenchymal transition” (EMT) [152]. This program allows cancer cells to acquire invasive properties, resist apoptosis, and disseminate to other tissues.

EMT is a physiological and pathological phenomenon that can plays a crucial role in the loss and gain of epithelial characteristics of cells, essential in breast cancer cell invasion and metastasis [153]. In the absence of EMT, breast cancer cells are columnar or cuboidal in shape, more polar, form tight cell–cell junctions, and remain stationary. However, when stimulated by external factors such as growth factors and cytokines, breast cancer cells can undergo EMT, transitioning into cells with a mesenchymal phenotype lacking polarity and gaining motility. During this EMT process [153], breast cancer cells gradually acquire a spindle-like fibrous shape, lose cell polarity, exhibit weak cell adhesion, become more loosely packed, and become prone to metastasis. EMT in breast cancer is a complex and highly regulated process [154]. Transcription factors, including zinc finger transcription factors, cell transformation regulators, E-box binding zinc finger proteins, and signaling pathways such as TGF-β, MAPK, and NF-κB, play a role in inducing and regulating EMT [155]. Activation of these transcription factors and signaling pathways can induce alterations in the biological characteristics of breast cancer cells, mainly including loss of adherens junctions and transition from polygonal/epithelial to elongated/fibroblastic morphology, expression of matrix-degrading enzymes, increased cell motility, and enhanced resistance to cell death. These biologically tailored alterations all contribute to breast cancer cells' invasive and metastatic processes. Activation of these transcription factors and signaling pathways can directly repress the expression of EMT suppressors, including E-cadherin, leading to the loss of this key suppressor and promoting invasiveness. However, some active ingredients in ginsenosides, including Rg3, Rg1, and CK, could effectively prevent the occurrence and development of breast cancer by targeting the related genes and proteins of EMT.

The intervention of ginsenoside Rh1 [72] on TNBC cells can affect the migration and invasion mediated by STAT3 and NF-κB signaling through the regulation of mtROS and downregulated the expression of metastasis factors (e.g., MMP2, MMP9, and VEGF-A), thus exerting a powerful anti-cancer effect. In MDA-MB-231 cells, the intervention of Rg1 [65] downregulated invasion and angiogenesis markers and modulated EMT markers, controlling breast cancer progression. As the most intensively studied active ingredient in the field of ginsenosides against breast cancer, Rg3 [95, 113, 156] reportedly inhibits breast cancer cell invasion by modulating immune activity, altering breast cancer stem cell phenotype, and affecting non-coding RNA expression after intervening in breast cancer cells. Some other rare ginsenoside components, such as 0(S)-Rh2 [157], Rd [158], CK [159], and 20(S)-PPD [160], can inhibit EMT in breast cancer cells through multiple targets and mechanisms. These studies suggest that EMT inhibition is also an important mechanism by which ginsenosides exert therapeutic effects anti-breast cancer.

### Ginsenosides reduce adverse effects from other medical treatments for breast cancer

The current standard treatment for breast cancer which is based on chemotherapy, radiotherapy, and targeted therapy, can cause damage to normal tissue organs while killing breast cancer cells. One of the main adverse reactions caused by these standard treatments is myocardial cell damage [161]. Anthracyclines are commonly used chemotherapy drugs for breast cancer, with significant cardiotoxicity, including cardiac dysfunction and heart failure [162]. These adverse effects may contribute to the inability of breast cancer patients to use adequate drug doses during treatment, rendering breast cancer a higher recurrence risk [163]. On the other hand, treatment with chemotherapeutic and targeted agents has the potential to cause drug resistance in breast cancer cells, leading to the failure of subsequent anti-breast cancer treatment.

At present, although the specific mechanism of action of anthracyclines leading to cardiotoxicity is not well defined, it has been established that the mechanism of injury mainly involves chelation of iron ions by anthracyclines, generation of reactive oxygen species that are mainly hydroxyl radicals and induction of lipid peroxidation in cardiomyocytes and mitochondrial damage in the heart machine [164]. On the other hand, the cardiotoxicity associated with anti-HER2 therapy is primarily attributed to the high expression of HER2 in cardiomyocytes. HER2 plays a crucial role in maintaining cardiomyocyte function and repairing cardiomyocyte damage. When anti-HER2 therapy blocks HER2 signaling, intracellular reactive oxygen species accumulate in cardiomyocytes, leading to injury and apoptosis [165]. Therefore, cardiotoxicity from medical treatment of breast cancer is mainly due to reactive oxygen species accumulation in cardiomyocytes due to treatment [166]. Ginsenosides, when combined with standard treatments, have shown the potential to inhibit adverse effects from standard treatments and prevent them.

Treatment with trastuzumab, an anti-HER2 therapy, can induce apoptosis in cardiomyocytes by upregulating the expression of apoptotic genes such as caspase-3, caspase-9, and Bax. However, ginsenoside Rg2 has been shown to reduce the expression of these apoptotic genes and attenuate trastuzumab-induced apoptosis in cardiomyocytes Rg2 [167]. Interestingly, Rg2 [168] induced protective autophagy in cardiomyocytes to avoid the apoptotic damage caused by trastuzumab by upregulating p-Akt, p-mTOR, Beclin 1, LC3, and ATG5 expression levels. As previously mentioned, doxorubicin can lead to cardiotoxic effects when treating breast cancer. Since doxorubicin causes histopathological changes, apoptosis and necrosis, and subsequent inflammation in the heart when treating breast cancer, Rh2 can reverse these changes [169]. Rh2, when administered with doxorubicin, alters the cell cycle and microtubule attachment, promotes TNF, chemokine, and interferon production, responses to cytokines and chemokines, and genes involved in T-cell activation, enhancing the efficacy of doxorubicin. In contrast, Rg1-conjugated [170] nanoparticles with doxorubicin can specifically bind to breast cancer cells to induce apoptosis and activate ROS and caspase-3 expression of tumor cells without damage to cardiomyocytes. Paclitaxel resistance is a common mechanism to reduce the benefit of paclitaxel treatment in breast cancer treatment. However, intervention with Panaxatriol can target paclitaxel-resistant breast cancer cells by blocking the activation of IRAK1/NF-κB and ERK signaling pathways, downregulating the expression of S100A7/9, inflammatory factors (IL-6, IL-8, CXCL1, CCL2), and cancer stem cell-related markers (OCT4, SOX2, NANOG, ALDH1, CD44), and restoring the sensitivity of breast cancer cells to paclitaxel [49].

## Ginsenoside has good anti-cancer activity in clinical application

At present, only four clinical studies on ginsenoside in cancer patients have been reported, including two studies on patients with non-small cell lung cancer [50, 171], one study on hepatocellular carcinoma [51] and one study on breast cancer [52]. In a randomized controlled study of ginsenoside Rg3 combined with capecitabine in the treatment of triple negative breast cancer [52], 30 patients with stage IV triple negative breast cancer received capecitabine alone (15 cases) or Rg3 combined with capecitabine (15 cases). Rg3 was administered twice a day (40 mg/day) for at least 2 weeks. The results showed that compared with chemotherapy with capecitabine alone, Rg3 + Capecitabine could achieve a higher short-term effective rate (*P* < *0.05*). Patients treated with Rg3 can also get lower incidence of adverse reactions and higher quality of life. This proves that ginsenoside combined with chemotherapy is a very potential treatment mode for patients with triple negative breast cancer.

In the study of Rg3 combined with chemotherapy in the treatment of stage II-III non-small cell lung cancer [50], 133 patients received Rg3(43 cases), Rg3 + chemotherapy (46 cases) or chemotherapy alone (44 cases). Rg3 was administered twice a day (40–50 mg/day) for at least 6 months. The results showed that compared with Rg3 or chemotherapy alone, Rg3 + chemotherapy improved the 3-year survival rate (54.3% versus 46.5% or 47.7%, respectively; *P* > 0.05). In the course of treatment, patients receiving Rg3 therapy have lower incidence of adverse reactions and better immune system function (increased activity of NK cells and CD4^+^ T cells and normal CD4^+^/CD8^+^ T cell ratio), which indicates that the combination of Rg3 therapy and chemotherapy is a very potential treatment strategy. In another study of Rg3 combined with TKI in the treatment of patients with unresectable EGFR mutation NSCLC in stage III–IV [171], 124 patients were divided into TKI + Rg3 (20 mg/day) or TKI monotherapy, and the administration lasted for at least 6 months. The results showed that Rg3 increased the median progression-free survival time of patients by 2.5 months, delayed acquired TKI drug resistance, and reduced TKI drug-related adverse reactions. Among the treatment-related adverse reactions, rash is the worst side effect in the two groups, while nausea, diarrhea and anorexia are the most common side effects in the two groups. However, the use of Rg3 reduces these adverse reactions caused by TKI drugs.

In another study of Rg3 in the treatment of patients with advanced hepatocellular carcinoma [51], 228 patients were randomly divided into two groups and received TACE alone or combined with Rg3 (40 mg/day). The results showed that the median overall survival time of patients receiving TACE + Rg3 was longer than that of patients receiving TACE alone (13.2 months compared with 10 months; *P* = 0.002). During the treatment, Rg3 was well tolerated, and the reported adverse effects were grade 1 or grade 2 constipation, nosebleed and hypertension. More importantly, the treatment of Rg3 is helpful to alleviate the adverse effects caused by TACE treatment and improve the quality of life of patients.

Although there are not many clinical studies on the treatment of cancer patients at present, it can be seen from the published studies that ginsenoside has a very bright prospect in the treatment of malignant patients. It has been found that ginsenoside can treat breast cancer through multi-targets, multi-channels and multi-mechanisms in vivo and in vitro, and the next stage of research should focus on transforming these research results into clinical use.

## Discussion

The fundamental hallmarks of a tumor are the defining characteristics that differentiate normal mammary epithelium from breast cancer cells. They are the underlying causes and driving forces behind the development and progression of breast cancer. These hallmarks of breast cancer cells, when combined, promote continuous proliferation, invasion, and metastasis. They also aid in the survival of breast cancer cells during conventional treatment methods and the acquisition of drug resistance. As a result, targeting breast cancer therapeutics modifies the basic hallmarks of breast cancer cells, causing them to lose the crucial features on which they depend for survival.

Therapeutic approaches and drugs targeting the general characteristics of tumor cells have shown success in breast cancer treatment. Ongoing basic research focused on tumors has led to the discovery of new fundamental hallmarks of tumors over the years. Therefore, therapeutic drugs and approaches that target these novel hallmarks hold significant potential in breast cancer treatment. In clinical breast cancer treatment, the commonly used therapeutic modalities aim to induce apoptosis (programmed cell death), block self-sufficient growth signals in cells, alter the ability of cells to replicate indefinitely, and enhance immune cells' recognition and killing of tumor cells, among other strategies [172]. However, both traditional and novel antitumor drugs may have side effects that can reduce patients’ quality of life and increase the risk of treatment resistance [161]. Natural compounds, which are naturally occurring antitumor agents, offer an alternative approach. By identifying potential antitumor drugs from informative data containing natural compounds, these compounds have the potential to be a class of antitumor therapeutics with fewer side effects, significant therapeutic benefits, and relative affordability [9].

Radix Ginseng, a widely utilized natural medicine, has been recognized for its therapeutic effects in modern pharmacological studies. It has demonstrated positive outcomes in anti-inflammation, antioxidant activity, antitumor properties, and energy supplementation [173]. Among the active components of Radix Ginseng, ginsenosides have been identified as the most predominant and abundant compounds, exhibiting significant potential in antitumor therapy [17]. Over 180 ginsenosides have been discovered so far, and most of these active components have demonstrated varying degrees of inhibition against breast cancer cells [174].

Ginsenosides, the primary active components with anticancer properties found in Radix Ginseng, are typically administered orally. However, some ginsenosides require metabolic transformation by gut microbes after oral intake to generate ginsenosides with enhanced anticancer activity [175]. This metabolic conversion primarily involves the conversion of protopanaxadiol-type ginsenosides into compound K and ginsenoside Rh2, as well as the conversion of protopanaxatriol-type ginsenosides into ginsenosides Rh1 and protopanaxatriol. Interestingly, ginsenosides can also influence the characteristics of breast cancer cells by modulating the species and abundance of intestinal flora and the levels of various metabolites in breast cancer patients. This modulation can have both attenuating and enhancing effects on breast cancer treatment [176].

Ginsenoside has been proved to have great potential in the treatment of breast cancer, but at present, the mainstream research direction is still ginsenoside combined with anti-tumor drugs (chemotherapy drugs, targeted therapy drugs, immunotherapy drugs, antibody-coupled conjugate), which proves that ginsenoside can enhance the efficacy of anti-tumor drugs. At present, many studies have confirmed that ginsenoside can delay the acquired drug resistance of tumor cells, but there is no relevant report on the drug resistance of tumor cells to ginsenoside. As a natural product, ginsenoside may acquire drug resistance in tumor cells for a longer time than other synthetic chemicals. At present, there is no research on the contraindications of ginsenoside in clinical use in the reports. Therefore, in the next research, the contraindications of ginsenoside in the treatment of malignant tumor patients should be clarified to facilitate the clinical use of ginsenoside.

Many studies have established that ginsenosides yield heterogeneous effects in breast cancer treatment, altering the key hallmarks of breast cancer cells, including apoptosis, tumor angiogenesis, EMT, and epigenetic changes [174]. While basic research has demonstrated the anti-breast cancer effects of ginsenosides, clinical research in this area is still ongoing [177]. Clinical studies have mainly focused on reducing toxic side effects and improving the efficacy of ginsenosides combined with anti-tumor drugs. Novel studies have also been conducted to improve the solubility and stability of ginsenosides in vivo by changing the carriers and modes of administration of ginsenosides [178]. Therefore, the application of ginsenosides in the treatment of breast cancer represents a promising strategy that has the potential to make significant breakthroughs in the future.

Ginsenoside has been proved to have great potential in the treatment of breast cancer, but at present, the mainstream research direction is still ginsenoside combined with anti-tumor drugs (chemotherapy drugs, targeted therapy drugs, immunotherapy drugs, antibody-coupled conjugate), which proves that ginsenoside can enhance the efficacy of anti-tumor drugs. At present, many studies have confirmed that ginsenoside can delay the acquired drug resistance of tumor cells, but there is no relevant report on the drug resistance of tumor cells to ginsenoside. As a natural product, ginsenoside may acquire drug resistance in tumor cells for a longer time than other synthetic chemicals. At present, there is no research on the contraindications of ginsenoside in clinical use in the reports. Therefore, in the next research, the contraindications of ginsenoside in the treatment of malignant tumor patients should be clarified.

## Data Availability

Not applicable.

## Abbreviations

Akt: Protein kinase B
AMPK: Adenosine 5′-monophosphate (AMP)-activated protein kinase
CAF: Cancer-associated fibroblast
DTX: Docetaxel
EMT: Epithelial–mesenchymal transition
ER: Estrogen receptor
HER2: Human epidermal growth factor receptor 2
IL: Interleukin
lncRNA: Long non-coding RNA
NF-κB: PINuclear factor kappa-B
MAPK: Mitogen-activated protein kinase
mTOR: Mammalian target of rapamycin
miRNA: Micro RNA
MMP: Matrix metallo proteinase
MDSC: Myeloid-derived suppressor cells
PR: Progesterone receptor
PI3K: Phosphatidylinositol-3 kinase
PD-1: Programmed cell death protein 1
PDGF: Platelet derived growth factor
ROS: Reactive oxygen species
STAT: Signal transducer and activator of transcription
TNBC: Triple negative breast cancer
TNF-α: Tumor necrosis factor-α
TME: Tumor microenvironment
VEGF: Vascular endothelial growth factor
